# Evaluation of Liquid and Bait Insecticides against the Dark Rover Ant (*Brachymyrmex patagonicus*)

**DOI:** 10.3390/insects5040832

**Published:** 2014-11-04

**Authors:** Javier G. Miguelena, Paul B. Baker

**Affiliations:** Department of Entomology, University of Arizona, 1140 E South Campus (Forbes Bldg.) Room 410, Tucson, AZ 85721-0036, USA; E-Mail: pbaker@ag.arizona.edu

**Keywords:** dark rover ant, *Brachymyrmex*, fipronil, indoxacarb, imidacloprid, lambda-cyhalothrin, borax, ant bait

## Abstract

Dark rover ants (*Brachymyrmex patagonicus*, Mayr) are an exotic ant species native to South America that has recently spread through the southern US. We evaluated the residual activity of three liquid insecticides (indoxacarb, fipronil and lambda-cyhalothrin) as potential barrier treatments against these ants. The factors we considered include the use of a porous or non-porous surface, a short or long exposure time and the changes in insecticide activity after treatment during a 90 day period. We also tested the effect of baits containing three different active ingredients (imidacloprid, sodium tetraborate and indoxacarb) on colony fragments of this species for a 15 day period. Both lambda-cyhalothrin® and indoxacarb® resulted in high levels of ant mortality up to 90 days after application. The results of exposure to fipronil® resembled those from the control treatment. Application of insecticides on a porous surface and the shorter exposure time generally resulted in greater ant survival. Of the baits tested, only the imidacloprid based one decreased ant survival significantly during the evaluation period. Within three days, the imidacloprid bait produced over 50% mortality which increased to over 95% by the end of the experiment. Results from the other two bait treatments were not significantly different from the control.

## 1. Introduction

Dark rover ants (*Brachymyrmex patagonicus* Mayr, 1968) are an exotic ant species native to South America [1] and were first reported in 1978 in Louisiana as *Brachymyrmex musculus* [2]. This recently introduced invasive ant has increased its distribution range and is currently located in most of the southern United States and in spots in the arid southwest [3,4,5]. These small (approximately 2 mm in length), dark brown ants form colonies with the potential of including thousands of individuals. Their nests can be found both indoors and outdoors, and they are especially attracted to sugary foods. Workers of these species can often be seen on counter tops and walls, meandering about as they search for food. In Arizona, we have collected female and male alates from mid-April through early November. They are primarily a nuisance species since they are not harmful to structures. Pest control professionals report that control of this ant is a challenging task, especially since these ants commonly reappear after apparently being controlled. Due to their recent emergence, little is known about the susceptibility of this species to currently available management techniques and insecticides.

With the present study, we intend to identify commercial insecticide formulations that can be used as tools for the management of dark rover ants. For this purpose, we evaluated the residual activity of six commercially available products: three liquid insecticides and three baits. As an invasive species, dark rover ants might be highly abundant in urban environments [4]. As a result, techniques that prevent these ants from invading structures will likely be valuable for their management. One such technique is the use of liquid insecticides with long lasting residual activity to form chemical barriers around structures. Under laboratory conditions, we tested the formulations of three widely used liquid insecticides: indoxacarb, lamba-cyhalothrin and fipronil. Besides the effectiveness of these products, we also considered the effect of treating porous or non-porous surfaces, two different exposure times and the loss of residual activity over time.

The use of insecticidal baits is another fundamental tool for the control of structure invading ants. Typically, baits ingested by worker ants are later shared with the rest of the colony and their delayed toxic effects result in the death of both the foragers and the individuals within the nest. This mechanism makes ant baits especially useful when nests are difficult to locate and if ant colonies have more than one nest. Dark rover ants may establish nests in close proximity to one another [4], and their nests are particularly difficult to locate due to their small size and the erratic nature of their foraging behavior. Sugar based liquid baits are a logical choice for the management of this species since colonies in the field have been reported to show a preference for naturally derived sugary liquids [6]. For our bait trial, we considered three bait formulations containing different active ingredients: imidacloprid, sodium tetraborate and indoxacarb. All the baits tested are meant for use indoors and are designed for the management of sugar loving ants. 

## 2. Experimental Section 

### 2.1. Liquid Insecticide Study

#### 2.1.1. Experiment Set Up

We used two substrates: A porous untreated pine wood (*Pinus* sp*.*) and a non-porous ceramic tile, both of which were in the form of 15.2 cm × 15.2 cm panels placed inside clear plastic food containers (15.8 cm × 15.7 cm × 5.6 cm) (Sure fresh®, Greenbrier International, Inc., Chesapeake, VA, USA). Wood panels had a thickness of 2.54 cm. All panels were glued down to the surface with an instant adhesive (Super glue®, Super glue Co: Rancho Cucamonga, CA, USA) and non-toxic caulking (All-purpose Polyseamseal®, Westlake, OH, USA) was placed around the edges touching the side of the panels to prevent the ants from going under them. Fluon® (Bioquip: Rancho Dominguez, CA, USA), a slippery polymer, was painted on all sides of the containers to prevent the ants from escaping. 

Three widely used liquid insecticides were tested: Indoxacarb, fipronil and lambda-cyhalothrin. More detail on each of the insecticides used can be found in Table 1. In all cases, we used the commercially available formulations. The insecticide concentrations used were those mandated by the insecticide label, and they were 0.06%, 0.03% and 0.06% for indoxacarb, lambda-cyhalothrin and fipronil respectively. Likewise, the application rates used were defined using the label instructions. For indoxacarb and lambda-cyhalothrin, the label specifies a rate of 40.75 mL/m^2^. Given the area of our panels (0.023 m²), the amount to be applied was 0.941 mL, which we approximated to 1 mL. By a similar calculation, we decided to use 1.5 mL of fipronil solution per panel which is equivalent to the 61 mL/m^2^ prescribed on its label. The indoxacarb label also contemplates the use of application volumes that are 2 and 4 times greater than the minimum application rate. These are suggested for larger infestations or difficult control conditions, including porous surfaces. We also tested the effects of indoxacarb at these rates, which correspond to 2 and 4 mL per panel. Henceforth, the minimal application of indoxacarb is designated as indoxacarb (1X), while the higher rates are identified as indoxacarb (2X) or (4X). A control treatment was also included and received a spray of 1 mL of deionized water only.

**Table 1:** Details of the insecticide formulations assessed.

Active ingredient	Manufacturer	Brand name	Chemical class	Concentration applied (%)	Application rate
**Liquid insecticides**					
Fipronil	BASF, Florham Park, NJ, USA	Termidor® SC	Phenylpyrazole	0.06	61 mL/m^2^
Lambda-cyhalothrin	Syngenta, Greensboro, NC, USA	Demand® CS	Pyrethroid	0.03	41 mL/m^2^
Indoxacarb (1X)		Arilon®	Oxadiazine	0.06	41 mL/m^2^
Indoxacarb (2X)					82 mL/m^2^
Indoxacarb (4X)					164 mL/m^2^
**Gel and liquid baits**					
Imidacloprid	Bayer CropScience, Research Triangle Park, NC, USA	Maxforce® Quantum ant bait	Neonicotinoid	0.03	0.1 g replenished as necessary
Sodium tetraborate	Rockwell labs Ltd., North Kansas City, MO, USA	Intice ® rover ant bait	Boron compound	5	
Indoxacarb	Syngenta, Greensboro, NC, USA	Advion® ant gel	Oxadiazine	0.05	

(1X), (2X) and (4X) are used to designate the 3 label instructed application rates tested for the indoxacarb treatment.

Surfaces received sprays under a fume hood to the top of the materials and were placed on a bench to air dry for 24 h. Spraying was done with travel-sized spray bottles that had been previously tested to determine the amount of liquid equivalent to one spray. A different spray bottle was used for each treatment. Approximately 25 dark rover ants (mean = 25.85, SD = 11.63) were introduced to each panel and remained on the panel for the designated exposure time. The ants were transferred to the panels by lightly knocking them off from the surfaces they inhabit inside artificial laboratory nests. This technique decreases the accuracy in the number of ants obtained each time but substantially reduces the mortality resulting from transfer. All ants used were obtained from mature queen-right laboratory colonies that we keep for this kind of experiments. All colonies were raised from mated queens that were collected within the urban area of Tucson, AZ.

Tests were conducted on the laboratory bench at approximately 25 °C and 20%–25% RH. After exposure, the ants were removed and placed in clear Petri dishes (8.2 cm × 1.3 cm) for the duration of the evaluation period. The sides of these Petri dishes were also coated with Fluon®. Evaluations of *B. patagonicus* survival were made 4 h after removal and repeated at 1, 2, 3 and 5, days after exposure. This consisted on counting the number of live ants remaining in each Petri dish. During this time, ants were offered water in the form of a small vial (3.5 cm × 1.2 cm) stopped with a piece of cotton and filled with water. Food was provided in the form of a 20% honey-water solution soaking a small cotton ball placed inside the Petri dishes. We replenished this food every other day. 

There were two exposure times of 5 min and 30 min with five replications for each treatment/ surface/exposure time combination. The treated panels were allowed to age in the laboratory near a window and were used to repeat the test at 7, 14, 30, 60 and 90 days after insecticide application. This surface aging was intended to simulate the process of insecticide degradation that occurs with moderate weathering, such as is experienced by indoor surfaces. Due to the large numbers of ants needed, between four and six dark rover ant colonies were used for each repetition. 

#### 2.1.2. Statistical Analysis

We used a multiple regression analysis to estimate the effects of each of the factors considered on the percent ant survival. For this analysis, the percentage of ants alive at the end of the five day evaluation period was considered. Parameters tested included insecticide treatment, exposure time, surface used and age of the treatment. Interactions between these factors were also included in the model in order to consider specific aspects of the insecticidal activity of the products. The parameterization of this analysis was done in such a way that the parameter estimates obtained were calculated with respect to the mean ant survival for each categorical variable. This allowed us to retain non-significant factors in the model without affecting the overall results. A *post-hoc* Tukey’s HSD test was performed when appropriate to compare the effects of different levels of categorical variables or their interactions. The first set of ant survival observations, corresponding to a surface aged for 1 day, showed considerably high mortality for all treatments, including the controls. We suspected that rough handling of the ants had resulted in additional mortality. This data was excluded from the multiple regression analysis to prevent it from confusing its results. Instead, the results for 1 day after application were tested independently among treatments with a two sample t-test. 

We also performed one-way ANOVAs on the percent ant survival after five days for each treatment age (1, 7, 14, 30, 60 and 90 days) in order to better describe the change in insecticidal activity over time. A *post-hoc* Tukey’s HSD test was used when appropriate to distinguish differences between pairs of treatments. The statistical software used for all analyses was JMP 8.0 [7].

### 2.2. Bait Study

#### 2.2.1. Experiment Set Up

The experiment was carried out using dark rover ant (*B. patagonicus*) colony fragments that were obtained from our laboratory colonies. In our experience, queenless colony fragments of this species behave in a similar manner to small colonies and are capable of surviving independently of their parent colony for over a year (unpublished data). The colony fragments consisted of approximately 40 dark rover ant workers (mean = 38.17, SD = 14.42) that were established inside plastic boxes. As in the liquid insecticides study, the variability in the number of ants per replicate was the results of the method employed to transfer them. This consisted in knocking the ants off the surfaces from their original habitat into the new one, avoiding directly touching the ants to prevent injury to their soft bodies. 

We used medium sized (15.5 cm × 11.87 cm × 5.8 cm) plastic boxes (Tri State Plastic, Latonia, KY, USA) for the ant’s habitat. Previous to the beginning of the experiment, the inside walls of the boxes were coated with Fluon® and allowed to dry. A 1.6 cm hole was burned on one side at about half a centimeter from the bottom of the container with an electric soldering iron. This enabled a 15 cm long piece of Tygon® tubing (Saint-Gobain Performance Plastic Co., Clearwater, FL, USA) to be inserted. The tube was used to connect the main ant habitat to a 150 mm × 20 mm Petri dish (Falcon®, Corning Inc., Corning, NY, USA) that had also been coated with Fluon® and had a similar hole burned on its side. The space around the tube was sealed using a hot glue gun. In the experiment, the Petri dish was used as a foraging area where the ants could encounter the insecticide baits. 

The test comprised six replicates of each of three liquid or gel bait treatments plus a 20% honey-water solution for the control. The active ingredients of the insecticide baits assessed were indoxacarb, sodium tetraborate and imidacloprid. More specific information on each of the bait formulations is included in Table 1. Baits were located in plastic bait stations (8 cm long × 5 cm wide) (Maxforce® bait station, Bayer CropScience, Research Triangle Park, NC, USA) inside the Petri dish. Each application consisted of 0.1 g of the bait or the honey water solution. Baits were checked daily for hardness with a clean dissecting needle. If hard to the touch, another application was done in an empty well of the bait station.

All tests were conducted in a reach-in incubator (Model I-36LL, Percival Scientific Inc., Perry, IA, USA) set at 27 °C and 50%–60% RH. Ants were placed inside the habitats and were allowed to adjust for 48 h before the baits were introduced. For the duration of the experiment, the ants were offered water in the form of cotton stopped, glass test tubes (13 mm × 100 mm) filled with distilled water. Each colony fragment was fed twice a week with their habitual diet consisting of a drop of 20% honey-water and one half of an adult cinereous cockroach (*Nauphoeta cinerea*). Food and water were placed inside the plastic box. We provided food during the experiment in order to simulate realistic conditions in which the ants infesting a structure will have other food sources beside the insecticidal baits. 

Survival counts were made 3, 6, 9, 13 and 15 days after the tubes were connected enabling the ants to forage into the bait arenas. We also recorded the number of ants inside the Petri dish at each time interval in order to identify any trends in the preference of the ants for the baits. 

#### 2.2.2. Statistical Analysis

We calculated the percentage of live ants with respect to the total ant population in each box for every time interval. This value was used as the response variable in a multiple regression analysis in which we considered the effects of the different bait treatments and the number of days after initial application. Because we were considering repeated measurements over time, we included a random variable identifying each replicate and used a restricted maximum likelihood (REML) approach. To make interpretation easier, the parameterization of this analysis was done in such a way that the effect of each treatment was compared with the control. To compare the final results directly, we also performed a one-way ANOVA on the percent ant survival after 15 days of treatment. 

We also compared the percentage of live ants out of the remaining living population that was observed inside the bait-containing Petri dishes at each time point. Due to the lack of normality in the data, we used a series of Kruskal-Wallis non-parametric tests. Observations corresponding to time points when all the ants in a given colony fragment had died were excluded from this analysis to avoid interpreting high mortality as low interest by the ants. When significant differences were detected, we performed Bonferroni corrected pairwise Mann-Whitney tests to compare between specific pairs of treatments. The statistical software used for all analyses was JMP 8.0 (SAS Institute, 2009) [7].

## 3. Results

### 3.1. Liquid Insecticides

Our multiple regression analysis (Table 2) found significant differences among the insecticidal performance of the different treatments considered. Dark rover ants had the lowest survival after exposure to lambda-cyhalothrin, and indoxacarb at the (2X) and (4X) application rates (Table 3). Indoxacarb applied at the lowest label prescribed rate (1X) had a slightly lower effect against dark rover ants. Although the mean ant survival in the fipronil treatment was significantly lower than that of the controls, it was also considerably higher than that of the other insecticide treatments (Table 3). 

The two surfaces tested also had significantly different effects on dark rover ant survival (Table 2). In general, the mean ant survival was lower when the treated surface was tile. However, this effect was dependent on the treatment applied (Table 3). We observed lower survival on tile than on wood for ants exposed to lambda-cyhalothrin and indoxacarb. Conversely, dark rover ants had greater survival on tile in the control and fipronil treatments. 

**Figure 1:**
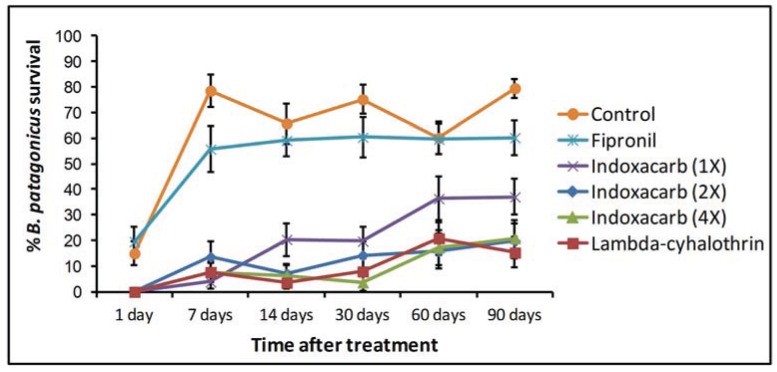
Change in insecticidal activity over time. This comparison is based on the mean ant survival at 5 days after exposure. Error bars were constructed with the standard error of the mean.

Mean dark rover ant survival showed a small but significant increase as the age of the applied treatments increased (Table 2, Figure 1). This result was not equal among treatments since the effect was slightly higher for indoxacarb at the lowest rate (1X) (Table 2). There was no significant interaction between the surface used and the age of the treatments (Table 2).

The amount of time that the dark rover ants were exposed to the treated surfaces also had a significant effect on survival. Ants exposed for 30 min had a lower mean survival than those exposed for 5 min (Table 2). We found no evidence of differences on this effect among treatments. 

**Table 2:** Parameter estimates and significance values for a multiple regression analysis (R^2^ = 0.55, F = 29.482, df = 24, 575, N = 600, *p* < 0.0001) on the percentage dark rover ant survival at 5 days after exposure to treated surfaces. The value of the parameter estimate for each level of the categorical variables and their interactions represents deviations from the mean dark rover ant survival of each of those variables. For categorical variables with two levels (surface and exposure time), one of the levels (wood and 5 min respectively) is not presented since its values are the same as that of the other level but with the sign of the parameter estimate inverted. This also applies to interactions of those variables.

Model parameters		Parameter estimates (± SE)	*p* > t
**Intercept**		33.25 ± 1.03	<0.0001*
**Treatment age**		5.4 ± 1.39	0.0001*
**Treatment**			
	Control	39.53 ± 2.3	<0.0001*
	Indoxacarb (1X)	−8.02 ± 2.3	0.0006*
	Indoxacarb (2X)	−17.91 ± 2.3	<0.0001*
	Indoxacarb (4X)	−21.91 ± 2.3	<0.0001*
	Lambda-cyhalothrin	−20.55 ± 2.3	<0.0001*
	Fipronil	28.86 ± 2.3	<0.0001*
**Treatment age × Treatment**			
	Treatment age × Control	−2.32 ± 3.1	0.4552
	Treatment age × Indoxacarb (1X)	8.6 ± 3.1	0.0058*
	Treatment age × Indoxacarb (2X)	−2.02 ± 3.1	0.5149
	Treatment age × Indoxacarb (4X)	0.29 ± 3.1	0.9252
	Treatment age × Lambda-cyhalothrin	0.18 ± 3.1	0.9546
	Treatment age × Fipronil	−4.73 ± 3.1	0.1284
**Surface**			
	Tile	−3.97 ± 1.03	0.0001*
**Surface × Treatment**			
	Tile × Control	10.07 ± 2.3	<0.0001*
	Tile × Indoxacarb (1X)	−3.42 ± 2.3	0.1394
	Tile × Indoxacarb (2X)	−7.11 ± 2.3	0.0022*
	Tile × Indoxacarb (4X)	−6.3 ± 2.3	0.0065*
	Tile × Lambda-cyhalothrin	−8.15 ± 2.3	0.0004*
	Tile × Fipronil	14.91 ± 2.3	<0.0001*
**Treatment age × Surface**			
	Treatment age × Tile	−1.73 ± 1.39	0.213
**Exposure time**			
	30 min	−2.97 ± 1.03	0.0041*
**Exposure time × Treatment**			
	30 min × Control	1.01 ± 2.3	0.6623
	30 min × Indoxacarb (1X)	−3.74 ± 2.3	0.1063
	30 min × Indoxacarb (2X)	−1.9 ± 2.3	0.4112
	30 min × Indoxacarb (4X)	−0.08 ± 2.3	0.971
	30 min × Lambda-cyhalothrin	2.06 ± 2.3	0.3717
	30 min × Fipronil	2.64 ± 2.3	0.2525

The multiplication sign (×) is used to designate interaction terms between parameters in the model. * indicates significance at the 95% level. The standard error of the mean is presented for each parameter estimate.

**Table 3:** Results of *post-hoc* Tukey’s HSD tests on significant treatment and interaction terms of our regression model for dark rover ant percent survival after exposure to insecticide treated surfaces. Levels not connected by the same letter are significantly different at the 0.05 level. The least square means of the *B. patagonicus* survival and their 95% confidence intervals are displayed.

Treatment						Surface × Treatment (interaction)		
Level	Contrast				LS Means (95% CI)	Level	Contrast	LS Means (95% CI)
Control	A				72.77 (67.8–77.74)	T × Control	A	78.88 (71.85–85.9)
Fipronil		B			62.11 (57.14–67.07)	T × Fipronil	A	73.05 (66.02–80.08)
Indoxacarb (1X)			C		25.23 (20.26–30.2)	W × Control	A B	66.67 (59.64–73.69)
Indoxacarb (2X)			C	D	15.33 (10.37–20.3)	W × Fipronil	B	51.16 (44.14–58.19)
Lambda-cyhalothrin				D	12.69 (7.72–17.66)	W × Indoxacarb (1X)	C	32.61 (25.59–39.64)
Indoxacarb (4X)				D	11.34 (6.37–16.31)	W × Indoxacarb (2X)	C	26.41 (19.39–33.44)
						W × Lambda-cyhalothrin	C	24.81 (17.79–31.84)
						W × Indoxacarb (4X)	C	21.61 (14.58–28.64)
						T × Indoxacarb (1X)	C D	17.84 (10.82–24.87)
						T × Indoxacarb (2X)	DE	4.26 (−2.77–11.28)
						T × Indoxacarb (4X)	E	1.07 (−5.96–8.1)
						T × Lambda-cyhalothrin	E	0.57 (−6.46–7.6)

For the Surface × Treatment levels, T = tile surface and W = wood surface

**Figure 2:**
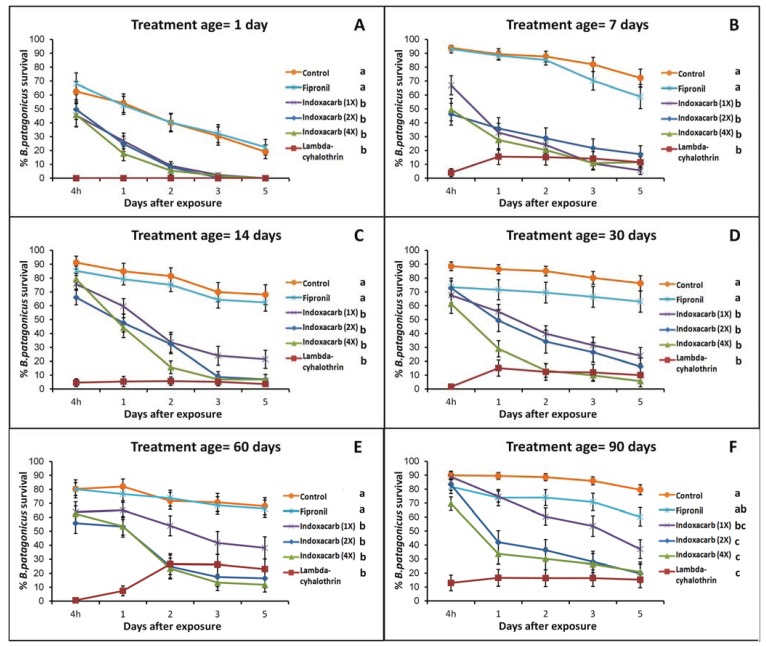
Mean percent ant survival under different insecticide treatments and using surfaces aged for different periods after application. Error bars were constructed with the standard error of the mean. Letters next to treatment names show the result of a *post-hoc* Tukey’s HSD test performed after one-way ANOVAs comparing the ant survival at 5 days after exposure. For the 1 day treatment age, a two sample *t*-test was used to compare the fipronil and control treatments instead since the survival at 5 days after exposure in the other treatments was zero for all replicates.

When the results from each treatment age were analyzed independently, we found significant differences among treatments in all cases (Figure 2). The pattern of these differences was the same at all evaluation times with the exception of the 90-day results. Before this time, lambda-cyhalothrin and indoxacarb at their different application rates produced high ant mortality while fipronil provided results that were indistinguishable from those in the control treatment. At 90 days, fipronil produced slightly higher mortality than in previous observations, but this was still not significantly different from the control treatment (Figure 2F). At the same time, the indoxacarb (1X) treatment produced a significantly lower mortality than the same insecticide at higher application rates (2X and 4X) and lambda-cyhalothrin. With the exception of the results at 1 day after application (Figure 2A), both the controls and the fipronil treated colony fragments had a final survival above 50% at all treatment dates. 

We observed lower ant survival across all treatments at 1 day after application, but this is likely attributable to rough handling in the transfer of the ants that was subsequently corrected. The highest mortality for any treatment was observed at that point. Lambda-cyhalothrin and the three application rates of indoxacarb resulted in 0% survival in that trial. In contrast, the ant survival in the fipronil treatment was not significantly different from that of the controls (two sample *t*-test, df = 38, t = 0.43, *p* = 0.6671). These results are somewhat suspect since the survival of the control was also low. Regardless of this, the results for that time point showed the same pattern with respect to the different treatments as those obtained in later dates.

The insecticides also differed in the way that survival changed after exposure (Figure 2). Indoxacarb, at its different application rates, produced relatively low mortality at 4 h after treatment, but this increased in the following days. By day 3, most of the affected ants were dead, there being little difference between the survival at 3 and 5 days after exposure. In contrast, with lambda-cyhalothrin some of the ants that seemed to be dead were eventually able to recover. Typically, these ants would be motionless and in contorted positions but would later regain full mobility. The highest apparent mortality occurred at 4 h after application, and recovery occurred in the following days. This recovery effect became more evident as the treated surfaces aged (Figure 2). Despite this, exposure to lambda-cyhalothrin consistently resulted in relatively high ant mortality at 5 days after exposure. By 90 days after application, the initial “knockdown” effect of lambda-cyhalothrin was not observed (Figure 2F).

### 3.2. Bait Study

**Figure 3:**
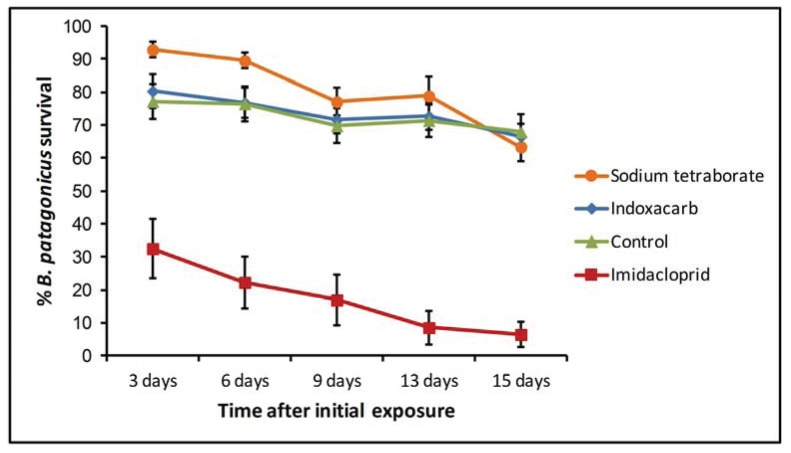
Changes in the mean percent *B. patagonicus* survival over time during their exposure to different bait treatments. Error bars were constructed with the standard error of the mean.

Our mixed effects multiple regression analysis (R^2^ = 0.93, N = 120) revealed that only the mean ant survival for the imidacloprid treatment was significantly different from that of the controls (Table 4) while the sodium tetraborate and indoxacarb treatments resulted in no significant effects. This analysis also revealed that in the indoxacarb treatment the change in the cumulative mortality over time was similar to that of controls. On the other hand, in both the sodium tetraborate and imidacloprid treatments, the dark rover ant mortality increased at a significantly greater rate than the controls (Table 4). This can be observed in the decrease in ant survival for the sodium tetraborate treatment between the 13 and 15 day evaluations and in the continuous decrease observed in the imidacloprid treatment over time (Figure 3). 

**Table 4:** Results of multiple regression analysis on the percentage dark rover ant mortality as a result of exposure to different bait treatments. Parameter estimates represent deviations from the results obtained from the control treatment.

Model parameters		Parameter estimates (± SE)	*p* > t
**Intercept**		79.44 ± 5.82	<0.0001*
**Time after initial application**		−0.75 ± 0.38	0.0489*
**Treatment**			
	Indoxacarb	1.07 ± 6.63	0.8736
	Sodium tetraborate	7.78 ± 6.63	0.2543
	Imidacloprid	−55.2 ± 6.63	<0.0001*
**Time after initial application × Treatment**			
	Time after initial application × Indoxacarb	−0.25 ± 0.53	0.6439
	Time after initial application × Sodium tetraborate	−1.42 ± 0.53	0.0087*
	Time after initial application × Imidacloprid	−1.38 ± 0.53	0.0107*

The multiplication sign (×) is used to designate interaction terms among parameters in the model. * indicates significance at the 95% level. Parameter estimates are followed by their standard errors.

**Figure 4:**
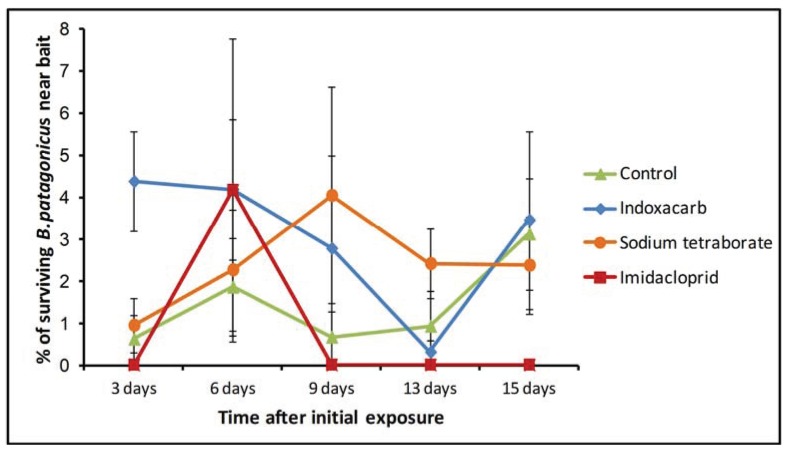
Mean percentage of surviving *B. patagonicus* found in the Petri dish containing each bait treatment at different times. Error bars were constructed with the standard error of the mean.

When the results at 15 days after initial exposure were considered, we found that only the imidacloprid bait produced a mean ant survival significantly lower from that of the control treatment (one way ANOVA, F = 38.27, df = 3,20, *p* = 0.0001).

Our analysis of the presence of dark rover ants in the Petri dishes containing the different baits revealed almost no significant differences among the bait treatments. The only exception was the 3 day evaluation point (Kruskal-Wallis test, df = 3, χ2 = 11.15, *p* = 0.011). Although a visual comparison of the results suggests that the indoxacarb treatment had a higher ant presence at that point (Figure 4), pairwise comparisons of all treatments were not significant. In this case, the small number of replicates per treatment might have prevented us from detecting significant differences. Importantly, some level of activity was observed in the sodium tetraborate, indoxacarb and control Petri dishes up to the end of the experiment (Figure 4). In contrast, no live ants were observed near the bait stations in the imidacloprid treatment after 9 days. It is relevant to consider that two of the ant colonies in the imidacloprid treatment were completely dead by the sixth day of the experiment, and two more were dead at the 13 day evaluation. 

By the end of the experiment, the two colonies in the imidacloprid treatment that remained alive had a mortality of 80 and 81.2%. None of the colonies in the other treatments experienced complete mortality by the end of the experiment.

## 4. Discussion

Our test of liquid insecticides revealed important differences in their capacity to kill off workers and potential efficacy against dark rover ants. While lambda-cyhalothrin and indoxacarb repeatedly produced high levels of mortality; fipronil had only a small effect on the ants. Given that fipronil is a broad spectrum insecticide that affects the insect nervous system, the low susceptibility of dark rover ants to it was unexpected. In previous studies, ants are usually highly susceptible to barrier treatments using this insecticide [8,9,10]. Variation in the susceptibility of ants to fipronil was reported by Hannum and Miller [11] who tested this insecticide against the black carpenter ant (*Camponotus pennsylvanicus*). They noted that there was considerable variation in susceptibility among different colonies, with the LT_50_ ranging from 7.4 to 29.3 h. However, after 40 h ants from all the colonies they considered reached a mortality of 100%. Another invasive species, the white-footed ant (*Technomyrmex difficilis*) has also shown reduced susceptibility to fipronil under laboratory conditions [12]. Mortality of white-footed ant colonies exposed to fipronil was estimated to reach 53% at 51 days after exposure. Besides that, residual treatments of fipronil against this species under field conditions did not result in mortality levels consistent with control [13]. 

We also found significant differences between the efficacies of insecticides depending on whether the treated surface was tile or wood. In general, ants exposed to the insecticides on tile had a greater mortality than those in the wood treatments. Several studies have found similar results in which insecticide treatment was more effective on a non-porous surface than on a porous one [9,14,15]. In the particular case of lambda-cyhalothrin, a study against the German cockroach (*Blattella germanica*) that included both unpainted plywood and vinyl tile as treated surfaces found that the non-porous surface resulted in 1 to 6% higher mortality at different times after application [16]. This can be explained because in a porous surface some of the insecticide can migrate below the surface and become unavailable. Interestingly, we also found a significant interaction between the effect of the surface used and that of the treatment applied. The control and fipronil treatments resulted in greater mortality on wood, while all other treatments produced greater mortality on tile. We believe that contact with the wood surface caused some mortality that was unrelated to the effect of the insecticides, possibly due to the roughness of the surface, which had the potential of causing physical injuries to ants during removal. In those treatments where the activity of the insecticides was absent or negligible (fipronil and control), this small mortality effect was evident. However, in the treatments where the action of the insecticides was the main source of mortality, the effect was masked by the non-porous quality of tile and its benefits to insecticide efficacy. 

As could be expected, a longer exposure time to the treated surfaces resulted in greater mortality. The magnitude of this effect was relatively small compared to other factors considered. We found no interaction between this effect and each particular insecticide treatment. Essentially, longer exposure reduced ant survival by a similar magnitude regardless of the treatment used. This includes the control treatment, which suggests that some of the additional mortality observed with longer exposure might not be the result of the insecticidal activity of the other treatments. Instead, the longer exposure itself might have been stressful to the ants in ways that induced mortality. For example, walking around on the treated surface for 30 min without access to water could have caused dehydration on the dark rover ants, thus increasing later mortality.

With respect to the loss of insecticidal activity over time, we found that as the treated surfaces aged, there was a small reduction in the mortality associated with them. This effect was uniform across treatments with the exception of the indoxacarb (1X) treatment which lost its insecticidal activity at a significantly higher rate. One of the practical implications of this outcome is that lambda-cyhalothrin has the capacity to retain most of its residual insecticidal activity for at least 90 days after application when used indoors. On the other hand, indoxacarb’s residual activity is somewhat reduced after 30 days at the lowest application rate (1X), but higher application rates can produce results similar to those observed with lambda-cyhalothrin. Our results also suggest that whether the treatment is applied to a porous or non-porous surface does not affect the rate of decrease of the residual activity over time. It is important to consider that the long residual activity observed in this experiment is particular to the use of the insecticides indoors. The same products being applied outdoors are likely to lose their efficacy considerably faster. 

The insecticides we tested showed differences in the progression of dark rover ant mortality after exposure. Exposure to lambda-cyhalothrin caused a fast knockdown effect, but some recovery was observed after day 1. The magnitude of the recovery increased as the treated surface aged. Although this implies a difference between the apparent and actual mortality after short exposure times, this difference might not be relevant in practice. Ants knocked down by lambda-cyhalothrin will normally stay on the treated surface and receive a new dose of the insecticide if they are able to recover and move. It is likely that recovery from knockdown was only made possible because we removed the ants from the treated surfaces. 

Exposure to indoxacarb resulted in a different progression of mortality which consisted in delayed toxicity. Mortality at 4 h after exposure was relatively low, but it increased for each new observation. Typically, the greater drop in survival occurred between the 4-hour and 2-day evaluation points. This result is important because it opens the possibility for transfer of the insecticide from exposed to unexposed ants through social interactions. However, there is currently no evidence of the capacity of indoxacarb to be transferred among ants when they are exposed to it by contact with a treated surface. Choe and Rust [17] found no significant transfer of indoxacarb among Argentine ants exposed to treated sand. On the other hand, there is evidence of the capacity of fipronil, to be transferred among ants after contact exposure [8,17]. On our experiment, the progression of mortality of ants exposed to fipronil closely resembled that of the ants in the control treatment. The overall low mortality in the fipronil treatment suggests that even if transfer could happen with this insecticide, it would have little effect on colony survival.

Our experiment with liquid and gel baits also found significant differences among the treatments considered. The imidacloprid bait produced the greatest ant mortality over time, while sodium tetraborate and indoxacarb had results that resembled those of the control treatment. In fact, the results of the imidacloprid treatment were the only ones that could be considered sufficient for the management of dark rover ants. Similar effects of treatments with liquid baits containing imidacloprid have been reported for the odorous house ant under field and laboratory conditions [18]. According to Rust *et al.* [19], who compared the characteristics of different formulations for the management of Argentine ants (*Linepithema humile*), imidacloprid shows delayed toxicity over a range of concentrations which makes it a suitable candidate for an active ingredient of baits used against ants. 

The limited mortality we observed in the indoxacarb treatment contrasts with previous studies that had found it to be generally effective for the management of structure invading ants. Indoxacarb based liquid baits have been reported to produce mortality at levels necessary for control in the red imported fire ant (*Solenopsis invicta*) [20], carpenter ant (*Camponotus modoc*) [21] and the Asian needle ant (*Pachycondyla chinensis*) [22]. To our knowledge, the only study that has described a relatively low mortality using an indoxacarb based bait was performed by Mathieson et al. [23]. They reported that in a 21 day laboratory trial of baits against the Argentine ant (*Linepithema humile*), an indoxacarb ant gel was unable to produce colony mortalities over 60%. Among the possible reasons that they considered for these results, they mentioned a lack of palatability of the bait since they rarely observed the ants feeding on it. In our bait trial, the indoxacarb bait had a performance that was indistinguishable from that of the controls, even though dark rover ants were frequently seen in the vicinity of the bait. These results could be explained by a preference of the dark rover ants towards the alternative food we provide instead of the indoxacarb bait. 

The treatment with the sodium tetraborate bait also failed to decrease ant survival in a significant way. Contrasting results were obtained in a study of the effects of bait insecticides on dark rover ants (*B. patagonicus*) by Keefer and Gold [24]. A boric acid bait and a sodium tetraborate bait were among the gel formulations they considered. They found that both products were associated with a mortality of over 80% after 11 days of exposure. The differences between their results and ours might be a consequence of the different carrier formulations used in the baits tested. 

Another possible factor affecting our results with the sodium tetraborate bait is that the high concentration of its formulation (5%) might be repellent to dark rover ants. Limited rejection towards boron based baits with concentrations above 1% has been recorded in the red imported fire ant (*Solenopsis invicta*) [25] and the Argentine ant (*L. humile*) [26]. In our trial, if such an effect was present, it might have been augmented by the presence of alternative food sources in the ants’ habitat. 

The presence of alternative food sources in our experiment likely had a strong effect on the effectiveness of the bait products tested. In a small preliminary study (n = 3 colony fragments) we carried out with the sodium tetraborate bait, in which we offered no alternative food to the dark rover ants, we obtained a mortality of 100% at two weeks after application (unpublished data). The relevance of this factor was also noted by Hansen [27], who described significant differences on the efficacy of bait treatments using a variety of products against carpenter ants in choice compared with no-choice trials. In general, she explained that the trials in which ants were offered an alternative food source produced lower mortality. Given the apparent importance of this effect, we consider that bait efficacy studies should include alternative food sources whenever they attempt to simulate pest management situations in which, despite cultural control methods, it is not possible to locate and remove all food sources for an invading ant colony.

The progression of ant mortality was different among bait treatments in our bait trial. Ants exposed to the sodium tetraborate bait showed no obvious effect of the treatment 3 days after application, but their survival declined afterwards at a slightly faster rate than that observed for either the control or indoxacarb treatments. This is suggestive of the delayed activity expected of ant bait formulations. However, this decrease was insufficient to generate greater mortality than that of the controls by the end of the experiment. On the other hand, most of the mortality observed in the imidacloprid treatment had already happened by the third day after application. The rate of decrease in the ant survival in that treatment was significantly greater than the one in the control treatment. Although the high mortality eventually accomplished by the imidacloprid bait is encouraging for its use against dark rover ants, the fast speed at which it acts could prevent it from being disseminated inside larger colonies under field conditions. Further research should determine with more precision how quickly the bait acts and if this speed is compatible with its transfer among colony members. Also, although these results show considerable differences in the toxicity of bait formulations for worker ants, it is still necessary to assess the effects of bait products on complete queenright colonies under field and laboratory conditions. 

When we considered the number of ants found in the proximity of each bait at different times, we found few significant differences. At 3 days after application, the indoxacarb bait had a greater number of ants in its Petri dishes than the other treatments. After that time, no other significant differences were observed. With this evidence, we cannot conclude that there is a greater overall preference for either of the treatments considered. However, this result might have been affected by the rapid mortality in the imidacloprid treatment. Since most of the ants exposed to that bait were dead by day 3, it is likely that the foraging activity in those colony fragments became affected to the point where further collection of the bait was severely reduced. 

## 5. Conclusions

We found important differences in the performance of the different products tested. Although further testing under field conditions is necessary, we consider that lambda-cyhalothrin and indoxacarb have high potential to perform as effective chemical barrier treatments. Some loss of insecticidal activity was observed as the surfaces treated with liquid insecticides aged. However, the higher application rates of indoxacarb (2X and 4X), as well as lambda-cyhalothrin produced significant dark rover ant mortality even after 90 days. In general, greater insecticide efficacy was observed when the treated surface was non-porous instead of porous. This effect might merit adjustments in the insecticide treatment regime used depending on what kind of surface is treated. 

Among the bait treatments considered, imidacloprid generated significantly higher mortality than sodium tetraborate and indoxacarb. Only the treatment with imidacloprid resulted in mortality that would be consistent for the management of dark rover ants under field conditions. 

## References

[1] Martínez J.J., Quirán E.M., Bachmann A.O. (2004). The neotropical genus *Brachymyrmex* Mayr, 1868 (Hymenoptera: Formicidae) in Argentina. Redescription of the type species, *B. patagonicus* mayr, 1868, *B. bruchi* Forel, 1912 and *B. oculatus* Santschi, 1919. Acta Zool. Mex..

[2] Wheeler G.C., Wheeler J. (1978). *Brachymyrmex musculus*, a new ant in the United States. Entomol. News.

[3] MacGown J.A., Forster J.A. (2005). A preliminary list of the ants (Hymenoptera: Formicidae) of Alabama, USA. Entomol. News.

[4] MacGown J.A., Hill J.G., Deyrup M.A. (2007). *Brachymyrmex patagonicus* (Hymenoptera: Formicidae), an emerging pest species in the southeastern United States. Fla. Entomol..

[5] Miguelena J., Baker P.B. Ruining your picnic: Prevalence of ants in urban parks in Tucson, AZ. the Proceedings of the National Conference on Urban Entomology.

[6] Robbins M., Miller T.E. (2009). Patterns of ant activity on *Opuntia stricta* (cactaceae), a native host-plant of the invasive cactus moth, *Cactoblastis cactorum* (Lepidoptera: Pyralidae). Fla. Entomol..

[7] (2008). JMP.

[8] Soeprono A.M., Rust M.K. (2004). Effect of delayed toxicity of chemical barriers to control Argentine ants (Hymenoptera: Formicidae). J. Econ. Entomol..

[9] Buczkowski G., Scharf M.E., Ratliff C.R., Bennett G.W. (2005). Efficacy of simulated barrier treatments against laboratory colonies of pharaoh ant. J. Econ. Entomol..

[10] Wiltz B., Suiter D., Gardner W. (2010). Activity of bifenthrin, chlorfenapyr, fipronil, and thiamethoxam against red imported fire ants (Hymenoptera: Formicidae). J. Econ. Entomol..

[11] Hannum C.D., Miller D.M. (2008). Intercolony variation in the black carpenter ant (*Camponotus pennsylvanicus*) (Hymenoptera: Formicidae) response to fipronil (0.06%) residues. Sociobiology.

[12] Warner J., Scheffrahn R.H. (2005). Laboratory evaluation of baits, residual insecticides, and an ultrasonic device for control of white-footed ants, *Technomyrmex albipes* (Hymenoptera: Formicidae). Sociobiology.

[13] Warner J., Scheffrahn R.H., Yang R.-L. (2010). Arboreal bioassay for toxicity of residual and liquid bait insecticides against white-footed ants, *Technomyrmex difficilis* (Hymenoptera: Formicidae). Sociobiology.

[14] Knight R.L., Rust M.K. (1990). Repellency and efficacy of insecticides against foraging workers in laboratory colonies of Argentine ants (Hymenoptera: Formicidae). J. Econ. Entomol..

[15] Osbrink W.L., Lax A.R. (2002). Effect of tolerance to insecticides on substrate penetration by formosan subterranean termites (Isoptera: Rhinotermitidae). J. Econ. Entomol..

[16] Wege P., Hoppe M., Bywater A., Weeks S., Gallo T. A microencapsulated formulation of lambda-cyhalothrin. the Proceedings of the Third International Conference on Urban Pests.

[17] Choe D.-H., Rust M.K. (2008). Horizontal transfer of insecticides in laboratory colonies of the Argentine ant (Hymenoptera: Formicidae). J. Econ. Entomol..

[18] Brooks M., Nentwig G., Gutsmann V. (2009). Elimination of a *Tapinoma melanocephalum* (hymenoptera: Formicidae) infestation using imidacloprid bait. Int. Pest Control.

[19] Rust M.K., Reierson D.A., Klotz J.H. (2004). Delayed toxicity as a critical factor in the efficacy of aqueous baits for controlling Argentine ants (hymenoptera: Formicidae). J. Econ. Entomol..

[20] Furman B.D., Gold R.E. (2006). Trophallactic transmission and metabolism of the active ingredient indoxacarb in Advion™ (Hymenoptera: Formicidae). Sociobiology.

[21] Hansen L.D. Indoxacarb as a management tool for carpenter ants. the Proceedings of The National Conference on Urban Entomology.

[22] Mo Y. (2013). Temporal food preference and effectiveness of selected bait products against *Pachycondyla chinensis* (Emery) (Hymenoptera: Formicidae). Master Thesis.

[23] Mathieson M., Toft R., Lester P.J. (2012). Influence of toxic bait type and starvation on worker and queen mortality in laboratory colonies of Argentine ant (Hymenoptera: Formicidae). J. Econ. Entomol..

[24] Keefer T.C., Gold R.E. Biology and management of the dark rover ant (Hymenoptera: Formicidae). the Proceedings of the National Conference on Urban Entomology.

[25] Klotz J.H., Vail K.M., Williams D.F. (1997). Toxicity of a boric acid-sucrose water bait to *Solenopsis invicta* (Hymenoptera: Formicidae). J. Econ. Entomol..

[26] Klotz J.H., Greenberg L., Amrhein C., Rust M.K. (2000). Toxicity and repellency of borate-sucrose water baits to Argentine ants (Hymenoptera: Formicidae). J. Econ. Entomol..

[27] Hansen L.D. Inconsistencies in the use of baits in field trials and comparison to laboratory trials with carpenter ants (Hymenoptera: Formicidae). the Proceedings of the Sixth International Conference on Urban Pests.

